# Harmful Cooperation in Relay-Assisted MIMO Under Imperfect CSI

**DOI:** 10.3390/s26134061

**Published:** 2026-06-26

**Authors:** Nikolaos Mouziouras, Constantinos T. Angelis, Andreas Tsormpatzoglou, Evangelos Spyrou

**Affiliations:** Department of Informatics & Telecommunications, Campus of Arta, University of Ioannina, 47150 Arta, Greece; kangelis@uoi.gr (C.T.A.); atsormpa@uoi.gr (A.T.); e.spyrou@uoi.gr (E.S.)

**Keywords:** cooperative MIMO, imperfect CSI, decode-and-forward relaying, harmful cooperation region, relay error propagation, reliability-aware relay activation, ZF detection, system robustness

## Abstract

This paper investigates the performance of cooperative multiple-input multiple-output (MIMO) systems under practical operating impairments, with particular emphasis on imperfect channel state information (CSI) and relay decoding errors. Although Decode-and-Forward (DF) relaying can provide diversity gains under ideal assumptions, these gains may significantly degrade in practical wireless environments affected by channel uncertainty. The analysis demonstrates that imperfect CSI introduces residual interference, leading to SINR saturation and BER error floors in the high-SNR regime. In cooperative systems, this degradation becomes more severe due to relay error propagation, where erroneously detected relay symbols introduce additional structured interference at the destination. Consequently, cooperative transmission may underperform direct MIMO communication within specific SNR operating regimes. To characterize this behavior, the concept of a harmful cooperation region is introduced, describing the operating regime in which the combined effects of CSI uncertainty and relay decoding errors render cooperation detrimental. To mitigate these limitations, a reliability-aware relay activation mechanism is proposed, enabling selective relay participation according to channel quality. By suppressing unreliable relay transmissions, the proposed approach significantly reduces error propagation and improves BER performance, particularly in the medium-to-high SNR regime. In addition, the impact of antenna scaling is investigated. The results reveal a robustness transition in which larger MIMO configurations exhibit improved resilience to CSI imperfections due to increased spatial diversity and improved channel conditioning. Overall, the findings demonstrate that cooperative transmission under imperfect CSI is inherently SNR-dependent and that robust system design requires the joint consideration of channel uncertainty, relay reliability, and system dimension.

## 1. Introduction

Cooperative multiple-input multiple-output (MIMO) systems have attracted considerable research interest as an effective approach for improving reliability and spectral efficiency in wireless communications. In emerging beyond-5G and 6G wireless networks, cooperative and distributed MIMO architectures are expected to play an increasingly important role in enabling reliable and adaptive wireless connectivity under challenging propagation conditions [1]. By exploiting spatial diversity through relay-assisted transmission, cooperative schemes such as Decode-and-Forward (DF) relaying are capable of providing substantial performance gains under favorable channel conditions.

Despite these advantages, most existing studies rely on the assumption of perfect channel state information (CSI), an assumption that is rarely satisfied in practical wireless environments. In realistic systems, channel estimation errors introduce uncertainty that affects both signal detection and relay operation. As a result, spatial multiplexing MIMO systems employing linear receivers such as Zero-Forcing (ZF) or MMSE experience residual interference that cannot be fully eliminated by increasing the transmit power, ultimately leading to performance saturation at high SNR [2,3].

The impact of CSI uncertainty becomes even more pronounced in cooperative transmission scenarios. In DF relaying, decoding errors occurring at the relay may be forwarded to the destination, introducing structured interference and additional performance degradation [4,5]. Although imperfect CSI, relay error propagation, and cooperative MIMO systems have each been extensively studied in the literature, their combined impact on cooperation reliability remains insufficiently understood, particularly under realistic relay-assisted transmission conditions and across different SNR operating regimes.

Motivated by this observation, the present work investigates the behavior of cooperative MIMO transmission under realistic CSI conditions and demonstrates that cooperation is not always beneficial.

In the context of this work, realistic CSI conditions refer specifically to practical impairments associated with channel estimation uncertainty and relay decoding errors, which are intentionally isolated in order to clearly characterize their impact on cooperative MIMO performance.

More specifically, the analysis reveals the existence of a harmful cooperation region, namely the SNR regime in which cooperative transmission underperforms direct MIMO communication due to the combined effects of CSI-induced interference and relay decoding errors.

To address this limitation, a reliability-aware relay activation mechanism is proposed, allowing relay participation only under sufficiently reliable channel conditions. By selectively suppressing unreliable relay transmissions, the proposed approach effectively mitigates error propagation and improves overall system performance compared to conventional always-on cooperative transmission.

Beyond relay reliability, the impact of system dimension is also investigated. The results reveal a robustness transition as the antenna configuration increases: while small-scale cooperative systems remain highly sensitive to CSI uncertainty, larger MIMO configurations benefit from increased spatial diversity and improved channel conditioning, thereby reducing the relative impact of channel estimation errors [6,7].

The importance of robust operation under CSI uncertainty becomes even more pronounced in large-scale and emerging MIMO architectures. In particular, recent studies have highlighted that channel estimation remains a critical challenge in near-field XL-MIMO systems envisioned for future 6G networks, where spherical wavefront propagation and polar-domain sparsity fundamentally alter conventional CSI acquisition mechanisms [8,9]. Unlike recent virtual and collaborative MIMO studies that primarily emphasize cooperation gains through virtual antenna expansion, spectral efficiency enhancement, and practical deployment feasibility, the present work focuses on a fundamentally different problem: the reliability limits of cooperative MIMO under realistic non-ideal conditions. Specifically, this work investigates the combined impact of imperfect CSI and relay decoding errors and identifies the operating conditions under which cooperation may become detrimental rather than beneficial.

The main contributions of this work can be summarized as follows:

- Identification of the harmful cooperation region under imperfect CSI.
- Analysis of the joint impact of CSI uncertainty and relay error propagation.
- Development of a reliability-aware relay activation strategy that mitigates harmful cooperation under imperfect CSI by selectively enabling relay participation according to source–relay link reliability.
- Robustness analysis across multiple antenna configurations.

The remainder of this paper is organized as follows. Section 2 presents the related work, system model and simulation framework. Section 3 analyzes the performance degradation caused by imperfect CSI and relay errors. Section 4 introduces the proposed reliability-aware relay activation mechanism and threshold optimization strategy. Section 5 investigates antenna scaling and robustness behavior. Section 6 discusses the main findings and system-level insights. Finally, Section 7 concludes the paper.

## 2. Related Work, System Model and Simulation Framework

### 2.1. Related Work

The impact of imperfect channel state information (CSI) on MIMO detection has been extensively investigated in the literature. In particular, linear receivers such as Zero-Forcing (ZF) and MMSE are known to experience significant performance degradation under channel uncertainty, since channel mismatch introduces residual interference that cannot be fully suppressed through linear equalization. Earlier studies demonstrated that imperfect CSI substantially reduces detection reliability in MIMO systems [10], while more recent works have explored robust and data-driven detection approaches aimed at improving performance under channel uncertainty [11,12]. Although these methods improve detection robustness, most existing studies primarily focus on performance enhancement rather than on the fundamental performance limitations imposed by imperfect CSI itself.

Beyond point-to-point MIMO detection, CSI uncertainty has also received considerable attention in cooperative and relay-assisted communication systems [13,14]. Previous studies have investigated the impact of imperfect CSI under various practical communication scenarios, including full-duplex relaying, RF/FSO systems, cognitive radio and SWIPT-based MIMO architectures [15,16,17,18,19]. These works consistently show that channel uncertainty degrades system performance; however, their analysis is typically centered on outage probability or capacity metrics, with limited emphasis on the interaction between CSI errors and relay decoding reliability [20].

In addition to conventional relay-assisted cooperative systems, recent research has expanded cooperative wireless communications toward virtual and collaborative MIMO paradigms based on cooperation among nearby user terminals, particularly in the context of beyond-5G and 6G wireless systems. In such architectures, multiple nearby devices collaboratively share or forward received signals to form virtual antenna arrays, effectively increasing the number of available antennas and enhancing spatial multiplexing capability. Recent studies on end-user-centric collaborative MIMO (UE-CoMIMO) have demonstrated that cooperation among personal devices can improve effective channel quality, diversity and achievable spatial multiplexing gains through virtual antenna expansion [21,22]. These architectures are increasingly recognized as promising enablers of user-centric wireless communication in future 6G systems.

Recent works have also investigated practical aspects of terminal-collaborative and distributed MIMO systems, including signaling overhead, user-terminal selection, energy-aware cooperation strategies and performance under practical non-ideal conditions. Collaborative uplink MIMO studies have shown that cooperation overhead and terminal selection significantly influence the achievable performance gain [23]. Recent studies have also begun exploring energy-aware user grouping and reliability-aware cooperation strategies for improving cooperation efficiency in terminal-collaborative MIMO systems, highlighting the importance of both resource management and reliable collaborative decision exchange [24,25]. In parallel, distributed MIMO studies have further highlighted the impact of practical system impairments and demonstrated robustness against multiple imperfections, while experimental demonstrations have confirmed the practical feasibility of high-frequency-assisted terminal collaboration under realistic operating conditions [26,27].

Despite these important advances, existing virtual and collaborative MIMO studies primarily treat cooperation as a performance-enhancing mechanism, with emphasis on capacity improvement, spectral efficiency, cooperation overhead reduction, resource management and implementation feasibility. In most existing studies, cooperation is implicitly assumed to be beneficial, while the possibility of performance degradation caused by cooperation itself is not explicitly investigated. In particular, existing virtual and collaborative MIMO studies do not explicitly characterize operating conditions under which cooperative transmission may become detrimental relative to direct MIMO communication.

Moreover, relay selection and selective relaying strategies have been widely investigated to improve diversity performance and mitigate unfavorable channel conditions [28,29,30,31]. The trade-off between cooperative diversity gain and transmission reliability has also been studied through diversity–multiplexing trade-off analysis in cooperative systems [32]. More recent research has further extended cooperative MIMO analysis toward increasingly realistic communication settings, including multi-relay cooperative networks, RIS-assisted MIMO systems with hardware impairments, and secure relay-assisted transmission under imperfect CSI [33,34,35]. Despite these advances, existing studies primarily focus on performance enhancement, robustness, or optimization and do not explicitly characterize operating regimes in which cooperation itself may become detrimental.

Consequently, despite the extensive literature on imperfect CSI and cooperative communications, the combined impact of CSI uncertainty and relay decoding errors in cooperative MIMO systems remains insufficiently understood. In particular, existing studies do not explicitly identify the SNR-dependent operating conditions under which Decode-and-Forward cooperation underperforms direct MIMO transmission.

Motivated by this gap, the present work investigates whether cooperative transmission remains beneficial under imperfect CSI and relay decoding errors. The analysis shows that harmful cooperation regions can emerge under practical operating conditions, where cooperative MIMO transmission degrades performance relative to direct communication due to the joint effects of CSI-induced interference and relay error propagation. To address this limitation, a reliability-aware relay activation mechanism is proposed, enabling selective relay participation according to channel conditions. Furthermore, the analysis is extended to multiple antenna configurations, revealing a dimension-dependent transition in robustness under imperfect CSI. A structured comparison of related works and their limitations is provided in Table 1, highlighting the lack of prior analysis on the joint impact of CSI uncertainty and relay decoding reliability.

### 2.2. System Model

**Figure 1:**
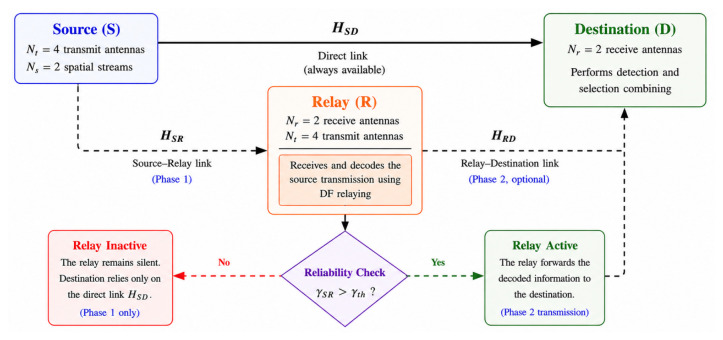
Architecture of the considered cooperative relay-assisted MIMO system with reliability-aware relay activation under imperfect CSI.

Figure 1 illustrates the considered cooperative relay-assisted MIMO communication framework. The system consists of a source (S), a relay (R) and a destination (D), with communication supported through both direct and relay-assisted transmission paths. Transmission follows a two-phase Decode-and-Forward (DF) protocol. During Phase 1, the source transmits simultaneously to both the relay and the destination through the source–relay and source–destination links, respectively. The relay then receives and decodes the transmitted signal and evaluates the reliability of the source–relay link using the proposed reliability-aware activation mechanism. If the source–relay link satisfies the reliability criterion, cooperative forwarding is activated during Phase 2 and the relay forwards the decoded information to the destination. Otherwise, relay participation is suppressed and communication relies solely on the direct source–destination link. All communication links are assumed to operate under imperfect CSI conditions.

The source is equipped with $N_{t}=4$ transmit antennas, while both the relay and the destination employ $N_{r}=2$ receive antennas.The system operates under spatial multiplexing with $N_{s}=2$ transmitted streams per channel use [6]. The transmitted symbol vector is defined as

(1) $$s\in C^{N_{s}\times 1},\;E\left[ss^{H}\right]=I$$

A linear precoder is employed in order to map the $N_{s}$ spatial streams onto the $N_{t}$ transmit antennas according to

(2) x=Fs

where

(3) $$F\in C^{N_{t}\times N_{s}}$$

denotes a semi-unitary precoding matrix satisfying

(4) $$F^{H}F=I$$

Thus, the transmitted vector satisfies

(5) $$x\in C^{N_{t}\times 1}$$

Transmission is performed in two phases following a Decode-and-Forward (DF) protocol [16,29].

Phase 1: Source Transmission: The received signals at the destination and the relay are, respectively, given by

(6) $$y_{\mathrm{SD}}=H_{\mathrm{SD}}Fs+n_{\mathrm{SD}}$$

(7) $$y_{\mathrm{SR}}=H_{\mathrm{SR}}Fs+n_{\mathrm{SR}}$$

where

(8) $$H_{\mathrm{SD}},H_{\mathrm{SR}}\in C^{N_{r}\times N_{t}}$$

and

(9) $$n∼\mathrm{CN}\left(0,\sigma_{n}^{2}I\right)$$

denotes additive white Gaussian noise (AWGN).

For notational simplicity, the effective channel matrices are defined as

(10) $$G_{\mathrm{SD}}=H_{\mathrm{SD}}F$$

(11) $$G_{\mathrm{SR}}=H_{\mathrm{SR}}F$$

(12) $$G_{\mathrm{RD}}=H_{\mathrm{RD}}F$$

where

(13) $$G_{ij}\in C^{N_{r}\times N_{s}}$$

For the considered $\left(N_{t},N_{r},N_{s}\right)=\left(4,2,2\right)$ configuration, the effective channel matrices become square matrices of dimension

(14) $$G_{ij}\in C^{2\times 2}$$

which enables linear ZF and MMSE detection.

Relay Decoding: The relay performs linear Zero-Forcing (ZF) detection based on the estimated effective channel matrix

(15) $${\hat{G}}_{SR}={\hat{H}}_{SR}F$$

The corresponding ZF detection matrix is expressed as

(16) $$W_{SR}={\hat{G}}_{SR}^{†}$$

where ${\left(\cdot \right)}^{†}$ denotes the Moore–Penrose pseudo-inverse.

The detected signal at the relay is therefore given by

(17) $${\hat{s}}_{R}=W_{SR}y_{SR}$$

Phase 2: Relay Transmission: During the second transmission phase, the relay forwards the detected signal to the destination according to

(18) $$y_{\mathrm{RD}}=H_{\mathrm{RD}}F{\hat{s}}_{R}+n_{\mathrm{RD}}$$

### 2.3. Channel Model

The channel matrices $H_{SD}$, $H_{SR}$, and $H_{RD}$ are assumed to follow independent Rayleigh fading with i.i.d. complex Gaussian entries [6].

Imperfect CSI is modeled as

(19) $$\hat{H}=H+E$$

where

(20) $$E∼\mathrm{CN}\left(0,\sigma_{e}^{2}I\right)$$

and the estimation error is assumed to be statistically independent of the true channel matrix H.

The corresponding estimated effective channel matrix is therefore expressed as

(21) $$\hat{G}=\hat{H}F$$

Substituting the imperfect CSI model into the received signal expression yields

(22) $$y=\hat{G}s+\left(G-\hat{G}\right)s+n$$

which reveals the presence of a residual interference term caused by channel estimation errors. Unlike thermal noise, this interference component does not vanish with increasing SNR, ultimately leading to performance saturation [3].

### 2.4. Receiver Processing

ZF Detection: At the destination, linear Zero-Forcing (ZF) detection is employed for both the direct and relay transmission links. Based on the estimated effective channel matrices, the corresponding ZF detection matrices are given by

(23) $$W_{\mathrm{SD}}={\hat{G}}_{\mathrm{SD}}^{†}$$

and

(24) $$W_{\mathrm{RD}}={\hat{G}}_{\mathrm{RD}}^{†}$$

The detected signals corresponding to the direct and relay-assisted transmissions are respectively expressed as

(25) $${\hat{s}}_{SD}=W_{SD}y_{SD}$$

(26) $${\hat{s}}_{RD}=W_{RD}y_{RD}$$

ZF detection is selected due to its simplicity and its ability to suppress inter-stream interference in spatial multiplexing systems [6,10]. However, under imperfect CSI, residual interference remains due to channel mismatch, which limits detection performance at high SNR.

Channel Gain-Based Selection Combining: At the destination, the final detected signal is selected according to the estimated effective channel quality of each transmission branch. The selection combining rule is expressed as

(27) $$\hat{s}=\left\{\begin{array}{cc} {\hat{s}}_{\mathrm{SD}},\; & \mathrm{if}\;\gamma_{\mathrm{SD}}\geq \gamma_{\mathrm{RD}}\\ {\hat{s}}_{\mathrm{RD}},\; & \mathrm{otherwise} \end{array}\right.$$

where

(28) $$\gamma_{\mathrm{SD}}={∥{\hat{G}}_{\mathrm{SD}}∥}_{F}^{2}$$

and

(29) $$\gamma_{\mathrm{RD}}={∥{\hat{G}}_{\mathrm{RD}}∥}_{F}^{2}$$

denote the estimated effective channel gains corresponding to the direct and relay-assisted transmission links, respectively.

The above formulation highlights two key aspects that critically affect system performance under realistic operating conditions. First, linear detection based on imperfect CSI introduces residual interference that cannot be fully eliminated, even at high SNR. Second, during the cooperative transmission phase, decoding errors at the relay may propagate to the destination, introducing an additional source of structured interference. The combined effect of these impairments plays a central role in the performance degradation analyzed in the following sections.

### 2.5. Simulation Setup

To evaluate the performance of the considered cooperative MIMO system, simulations were conducted using the Vienna System-Level Simulator (Vienna SLS), a widely adopted framework for wireless communication system evaluation. The use of a standardized simulation platform ensures reproducibility and enables consistent comparison with previously reported results in the literature.

Although Vienna SLS is primarily designed for system-level analysis, the present study focuses on link-level performance evaluation in order to isolate the effects of imperfect channel state information (CSI) and relay operation on the overall system behavior. In particular, the analysis emphasizes BER performance across different SNR regimes under realistic channel uncertainty conditions.

The baseline Vienna SLS configuration was adopted, with modifications restricted to the parameters relevant to the objectives of this work. More specifically, the antenna configuration, CSI error variance, and relay operation strategy were adjusted to investigate the impact of channel uncertainty and cooperative transmission under different operating conditions.

Unless otherwise specified, all remaining simulation parameters follow the default settings of the Vienna SLS environment. For reproducibility and transparency, the main simulation parameters used throughout this study are explicitly summarized in Table 2.

## 3. Performance Analysis

### 3.1. Interference-Limited Behavior Under Imperfect CSI

The previous sections established the system and channel models for cooperative MIMO transmission under imperfect CSI conditions. Building upon this framework, the present section analyzes the fundamental mechanisms responsible for the observed performance degradation at high SNR. In particular, the analysis focuses on the emergence of residual CSI-induced interference and relay error propagation, both of which progressively drive the system from a noise-limited regime toward an interference-limited operating regime.

Based on the imperfect CSI model introduced previously, the estimated channel matrix can be expressed as

(30) $$\hat{H}=H+E$$

where E denotes the channel estimation error matrix.

Considering the precoded transmission model, the corresponding estimated effective channel matrix becomes

(31) $$\hat{G}=\hat{H}F$$

where

(32) G=HF

denotes the true effective channel matrix.

Accordingly, the received signal can be written as

(33) $$y=\hat{G}s+\left(G-\hat{G}\right)s+n$$

where the second term represents residual interference caused by imperfect channel knowledge.

Substituting $\hat{G}=G+EF$, the received signal can be equivalently expressed as

(34) $$y=\hat{G}s-EFs+n$$

Using the ZF detection matrix

(35) $$W={\left({\hat{G}}^{H}\hat{G}\right)}^{-1}{\hat{G}}^{H}$$

the detected signal becomes

(36) $$\hat{s}=s-WEFs+Wn$$

where W denotes the ZF detection matrix.

The above expression reveals that the detection error consists of two distinct components: an amplified thermal noise term and a CSI-induced interference term. While the thermal noise contribution progressively decreases as the SNR increases, the interference component remains coupled to the transmitted signal power and therefore cannot be completely eliminated by increasing transmit power alone.

The residual CSI-induced interference term can be expressed as

(37) $$i_{\mathrm{CSI}}=WEFs$$

Accordingly, the effective post-detection SINR can be approximately expressed as

(38) $${\mathrm{SINR}}_{\mathrm{eff}}=\frac{P_{s}}{P_{\mathrm{CSI}}+P_{n}}$$

where $P_{s}$ denotes the desired signal power, $P_{\mathrm{CSI}}$ represents the residual interference power caused by imperfect CSI, and $P_{n}$ denotes the post-detection noise power.

As the transmit SNR increases, the thermal noise contribution progressively decreases, i.e.,

(39) $$P_{n}\to 0$$

whereas the CSI-induced interference component remains non-negligible:

(40) $$P_{\mathrm{CSI}}\neq 0$$

Assuming normalized transmit power and statistically independent estimation errors, the residual interference power after linear detection satisfies

(41) $$P_{\mathrm{CSI}}=E\left[{∥WEFs∥}^{2}\right]\propto \sigma_{e}^{2}P_{s}$$

which indicates that the CSI-induced interference scales proportionally with the transmitted signal power.

Consequently, in the high-SNR regime, the effective SINR approaches

(42) $$\lim_{\mathrm{SNR}\to \infty }{\mathrm{SINR}}_{\mathrm{eff}}=\frac{P_{s}}{P_{\mathrm{CSI}}}$$

revealing that system performance becomes dominated by residual interference rather than thermal noise.

This transition provides an analytical interpretation of the BER saturation and error-floor behavior observed in the simulation results. Since the CSI-induced interference power scales proportionally with the transmitted signal power, increasing the transmit power alone is insufficient to continuously improve BER performance under imperfect CSI conditions.

**Proposition** **1.** *Under imperfect CSI, the post-detection SINR of the considered linear MIMO receiver converges to a finite value in the high-SNR regime and therefore cannot increase unboundedly with transmit power.*

**Proof.**  From the detected signal model

(43) $$\hat{s}=s-WEFs+Wn$$

the effective detection error consists of a thermal noise term and a CSI-induced interference term. As the SNR increases, the thermal noise contribution progressively decreases. However, the interference term

(44) WEFs

remains proportional to the transmitted signal power due to the CSI error matrix E. Consequently, the interference power does not vanish in the high-SNR regime.

Therefore, the effective post-detection SINR converges to a finite value determined by the CSI error variance rather than increasing unboundedly with SNR. As a result, BER saturation and error-floor behavior appear under imperfect CSI conditions.    □

Beyond CSI-induced interference, practical Decode-and-Forward (DF) relaying systems are additionally affected by relay decoding errors, which introduce a second source of structured interference.

Due to imperfect relay detection, the decoded stream vector at the relay can be modeled as Due to imperfect relay detection, the decoded stream vector at the relay can be modeled as

(45) $${\hat{s}}_{R}=s+e_{R}$$

where $e_{R}$ denotes the relay decoding error vector.

The relay subsequently re-encodes and forwards the detected streams according to

(46) $$x_{R}=F{\hat{s}}_{R}$$

Thus, the received signal during the relay transmission phase becomes

(47) $$y_{\mathrm{RD}}=H_{\mathrm{RD}}F{\hat{s}}_{R}+n_{\mathrm{RD}}$$

Substituting the relay decoding model yields

(48) $$y_{\mathrm{RD}}=H_{\mathrm{RD}}Fs+H_{\mathrm{RD}}Fe_{R}+n_{\mathrm{RD}}$$

Using the effective channel definition

(49) $$G_{\mathrm{RD}}=H_{\mathrm{RD}}F$$

the received signal can be equivalently expressed as

(50) $$y_{\mathrm{RD}}=G_{\mathrm{RD}}s+G_{\mathrm{RD}}e_{R}+n_{\mathrm{RD}}$$

The second term represents structured interference introduced by relay decoding error propagation. Assuming that the relay decoding error probability remains non-zero under imperfect CSI conditions, the relay-induced interference term $G_{\mathrm{RD}}e_{R}$ also remains non-negligible in the high-SNR regime.

Consequently, cooperative DF systems operating under imperfect CSI are affected by compounded impairments, where residual CSI-induced interference and relay error propagation jointly contribute to BER saturation and high-SNR performance degradation.

**Figure 2:**
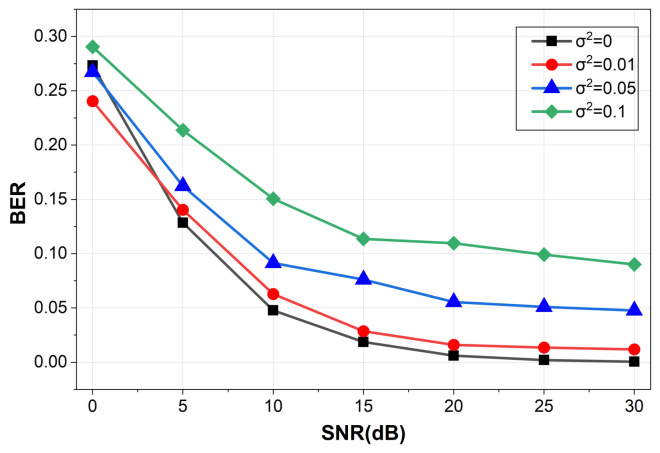
Relay decoding BER for the source–relay link under different CSI error variances.

This behavior is reflected in the relay decoding error rate illustrated in Figure 2, where a persistent BER floor can be observed under imperfect CSI conditions. As a result, the relay may continue forwarding erroneously detected symbols even when the direct transmission link operates under relatively favorable conditions.

**Figure 3:**
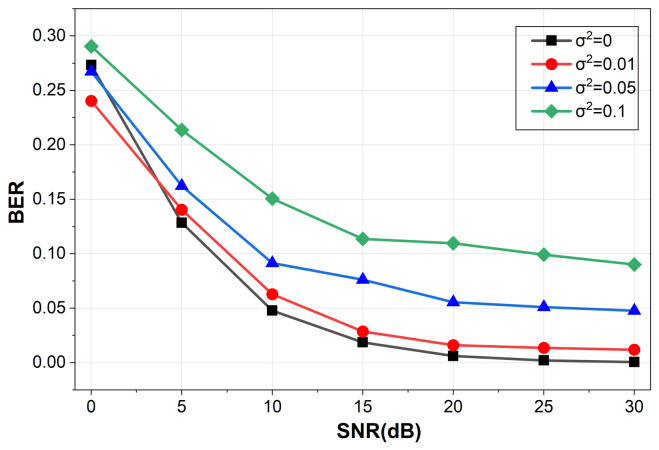
BER performance of cooperative DF MIMO with imperfect relay decoding under different CSI error variances.

The impact of relay error propagation on the overall system performance is illustrated in Figure 3. Compared to the ideal-relay scenario, the inclusion of relay decoding errors leads to substantially higher BER values together with a more pronounced error floor, particularly in the medium-to-high SNR regime.

Overall, the analysis demonstrates that cooperative MIMO systems operating under imperfect CSI progressively transition from noise-limited behavior toward interference-limited operation as the SNR increases. In this regime, residual CSI-induced interference together with relay error propagation fundamentally limits the achievable cooperative performance.

### 3.2. Direct and Cooperative MIMO Behavior Under Imperfect CSI

The performance of the baseline direct MIMO system is first evaluated under both perfect and imperfect channel state information (CSI) conditions in order to establish a reference point for the subsequent cooperative transmission analysis.

**Figure 4:**
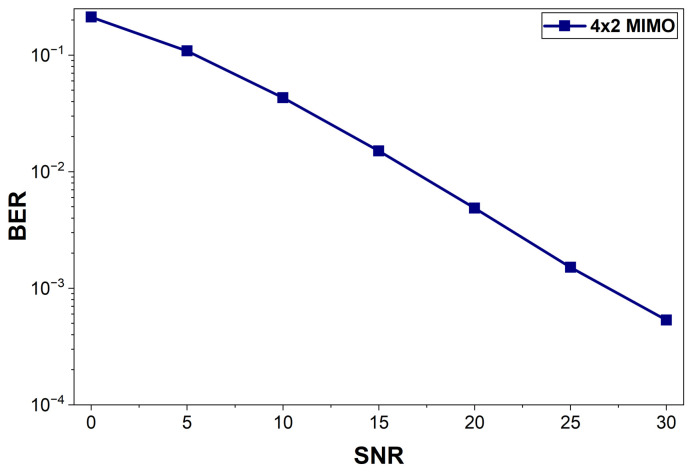
BER performance of direct 4×2 MIMO with ZF detection and perfect CSI.

Under perfect CSI, the direct 4×2 MIMO system exhibits the expected monotonic BER reduction with increasing SNR, as illustrated in Figure 4. The observed BER slope reflects the limited diversity gain provided by spatial multiplexing together with ZF detection [10].

**Figure 5:**
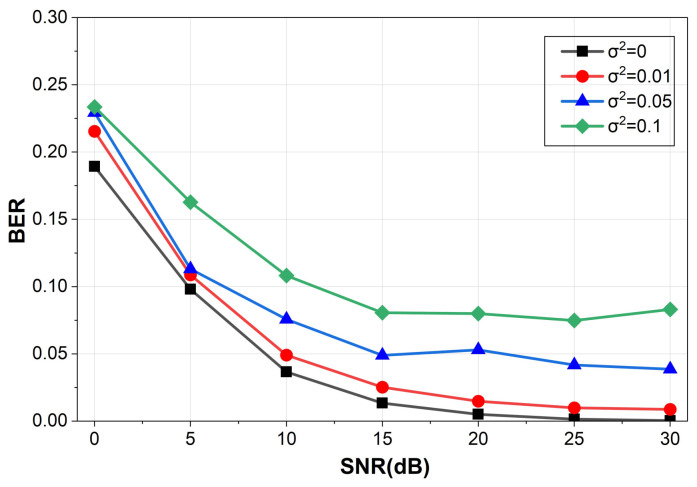
BER vs. SNR for direct 4×2 MIMO with imperfect CSI for different $\sigma_{e}^{2}$.

When imperfect CSI is introduced, however, the system behavior changes considerably. Although the degradation remains relatively limited at low SNR, a pronounced BER error floor gradually appears in the high-SNR regime, as shown in Figure 5. This behavior reflects the transition from a noise-limited operating regime to an interference-limited regime, where residual CSI-induced interference becomes dominant over thermal noise [3,10].

Consequently, increasing the transmit power no longer leads to continuous BER improvement, resulting instead in performance saturation. Moreover, the degradation becomes increasingly severe as the CSI error variance grows, highlighting channel estimation uncertainty as the dominant limiting factor under high-SNR conditions.

Having established the baseline behavior of direct transmission, cooperative DF relaying is next evaluated under ideal operating conditions assuming perfect CSI and error-free relay decoding [5]. This scenario provides an upper performance bound for cooperative transmission.

**Figure 6:**
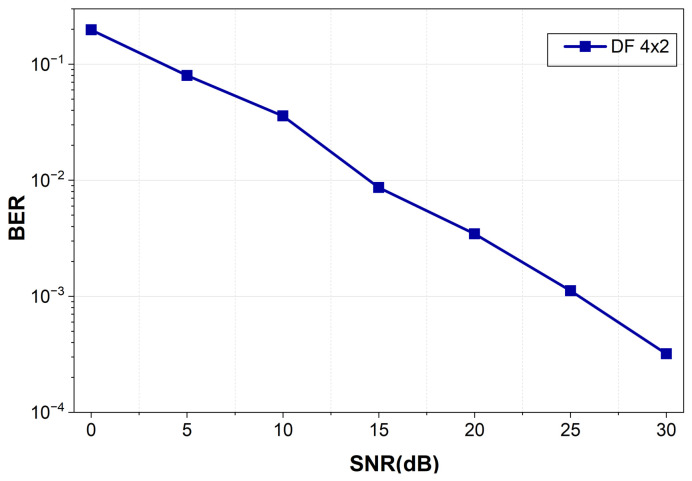
BER vs. SNR for cooperative DF (4×2) with ideal relay and perfect CSI.

As illustrated in Figure 6, cooperative transmission improves BER performance relative to direct MIMO transmission. Nevertheless, the achieved gain remains moderate due to the use of selection combining together with spatial multiplexing and ZF detection, which limits the achievable diversity order. As a result, the BER slope remains similar to that observed for direct transmission.

Most importantly, no BER error floor is observed under ideal operating conditions, confirming that the system remains noise-limited throughout the considered SNR range [3].

**Figure 7:**
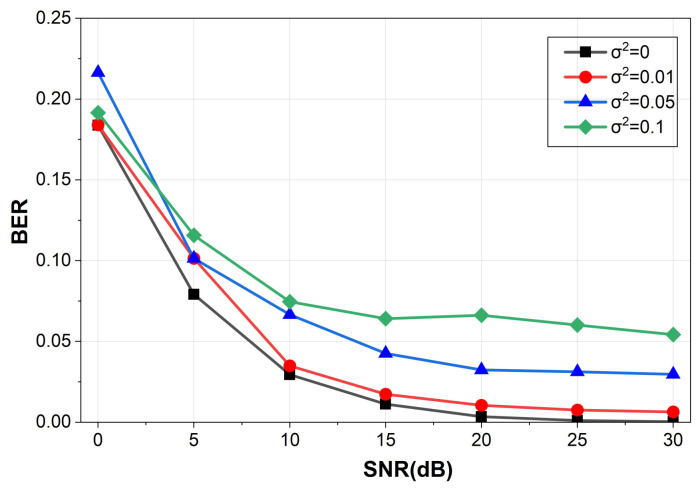
BER vs. SNR for cooperative DF (4×2) with imperfect CSI at the destination for different $\sigma_{e}^{2}$.

The behavior of the cooperative system changes significantly once CSI imperfections are introduced. Even when assuming ideal relay operation, a clear BER error floor emerges, as illustrated in Figure 7, indicating the onset of interference-limited behavior and the progressive reduction of effective diversity gain at high SNR.

This degradation becomes increasingly pronounced as the CSI error variance increases and follows the same interference-limited behavior previously observed in the direct MIMO case [3,10]. However, in cooperative transmission, the impact of imperfect CSI becomes more severe due to the additional sensitivity introduced by relay-assisted transmission together with relay error propagation.

**Figure 8:**
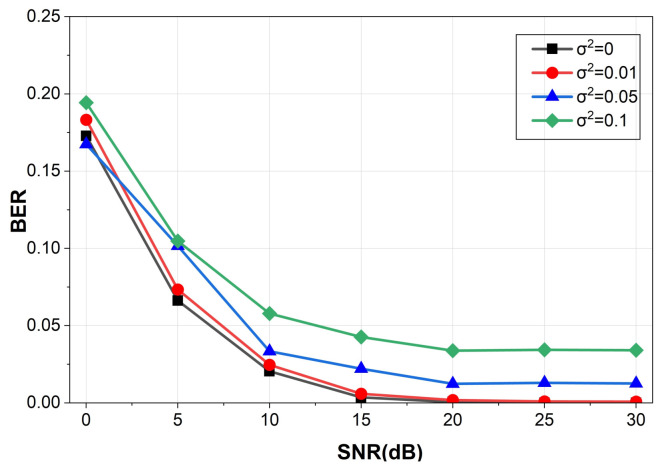
BER vs. SNR for cooperative DF (4×2) with imperfect CSI using MMSE detection for different $\sigma_{e}^{2}$.

Furthermore, this behavior is found to be largely independent of the specific linear receiver structure. As shown in Figure 8, MMSE detection improves BER performance in the low-to-medium SNR regime relative to ZF detection due to its improved noise-interference trade-off. Nevertheless, the high-SNR BER floor remains clearly visible, confirming that residual CSI-induced interference, rather than noise enhancement, constitutes the dominant performance limitation under severe CSI uncertainty.

Overall, the results demonstrate a progressive transition from noise-limited to interference-limited behavior as CSI uncertainty increases. While cooperative transmission provides diversity benefits under ideal operating conditions, its sensitivity to CSI mismatch and relay-induced interference substantially reduces the achievable performance gain under realistic CSI conditions.

### 3.3. Harmful Cooperation Region

The previous results demonstrated that the combined effects of CSI-induced interference and relay decoding errors can substantially reduce the benefits of cooperative transmission under practical operating impairments. To further characterize this behavior, the concept of a harmful cooperation region is introduced.

The harmful cooperation region is defined as the SNR range in which cooperative transmission underperforms direct MIMO communication, i.e.,

(51) $$\Delta \mathrm{BER}={\mathrm{BER}}_{\mathrm{coop}}-{\mathrm{BER}}_{\mathrm{direct}}>0$$

where ${\mathrm{BER}}_{\mathrm{coop}}$ corresponds to cooperative Decode-and-Forward transmission under imperfect CSI and imperfect relay decoding conditions.

Formally, the harmful cooperation region can be expressed as

(52) $$R_{\mathrm{harm}}=\left\{\mathrm{SNR}:{\mathrm{BER}}_{\mathrm{coop}}>{\mathrm{BER}}_{\mathrm{direct}}\right\}$$

From an effective SINR perspective, harmful cooperation behavior emerges whenever the effective SINR of the cooperative transmission becomes lower than that of direct MIMO transmission, i.e.,

(53) $${\mathrm{SINR}}_{\mathrm{coop}}<{\mathrm{SINR}}_{\mathrm{direct}}$$

Under imperfect CSI conditions, cooperative transmission is affected by both residual CSI-induced interference and relay error propagation. Consequently, the additional interference introduced by relay participation may exceed the achievable cooperative diversity gain, particularly in the high-SNR regime where interference becomes dominant over thermal noise.

Under imperfect CSI conditions, the behavior of the cooperative system changes significantly as CSI uncertainty increases. While cooperative and direct transmission exhibit comparable performance for low CSI error levels, their performance progressively diverges under stronger channel estimation errors.

**Figure 9:**
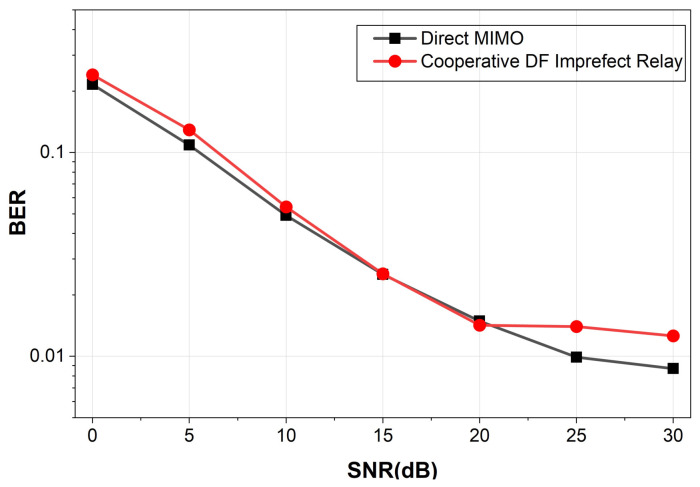
BER comparison of direct MIMO and cooperative DF with imperfect relay decoding for $\sigma_{e}^{2}=0.01$.

For moderate CSI error variance, the gains provided by cooperation gradually diminish and eventually reverse in the medium-to-high SNR regime, as illustrated in Figure 9. This behavior is primarily caused by the combined effects of residual CSI-induced interference and relay decoding error propagation [15].

**Figure 10:**
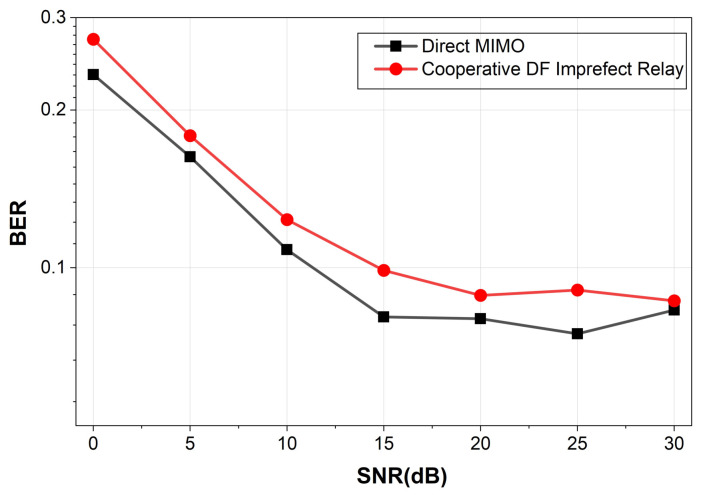
BER comparison of direct MIMO and cooperative DF with imperfect relay decoding for $\sigma_{e}^{2}=0.1$.

As the CSI uncertainty increases further, the degradation becomes even more pronounced, as shown in Figure 10, where cooperative transmission consistently underperforms direct MIMO over a wide SNR range. In this regime, the cooperative relay no longer improves link reliability, but instead introduces additional structured interference that propagates through the relay-assisted transmission path.

**Figure 11:**
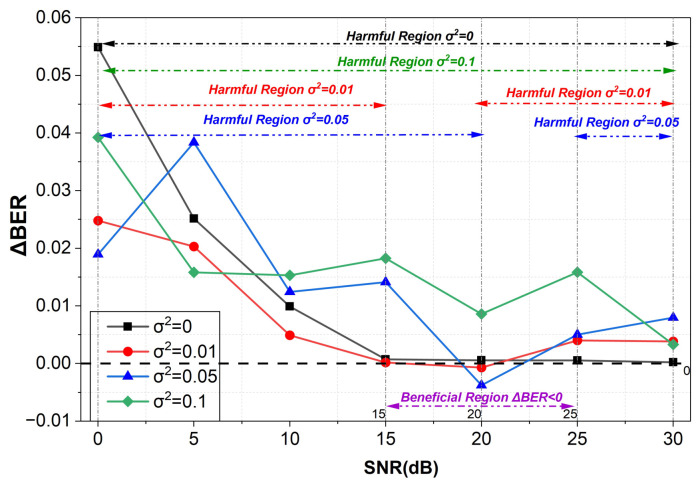
Harmful cooperation region, defined by $\Delta \mathrm{BER}={\mathrm{BER}}_{\mathrm{coop}}-{\mathrm{BER}}_{\mathrm{direct}}$, as a function of SNR for different CSI error variances $\sigma_{e}^{2}$. The corresponding SNR boundaries of the harmful region are explicitly identified in the figure. The horizontal dashed line indicates the boundary at ΔBER=0, separating harmful and beneficial cooperation regions.

The harmful cooperation region is explicitly illustrated in Figure 11, where positive values of

(54) $$\Delta \mathrm{BER}={\mathrm{BER}}_{\mathrm{coop}}-{\mathrm{BER}}_{\mathrm{direct}}>0$$

indicate that cooperative transmission becomes less reliable than direct MIMO communication.

As the CSI error variance increases, the harmful cooperation region progressively expands, demonstrating the increasing sensitivity of cooperative transmission to CSI uncertainty and relay decoding errors. These observations confirm that cooperative transmission is not inherently beneficial under realistic operating conditions and directly motivate the need for reliability-aware relay activation strategies.

It should be noted that the present analysis assumes statistically identical average channel conditions for all communication links and does not explicitly incorporate relay placement or distance-dependent path loss effects. This modeling choice was adopted in order to isolate the impact of CSI uncertainty and relay error propagation on cooperative MIMO performance. Consequently, the identified Harmful Cooperation Region reflects the effects of imperfect CSI and relay error propagation rather than a particular relay topology or placement configuration.

To further examine whether the harmful cooperation phenomenon is merely an artifact of uncoded BER evaluation, an additional coded-system validation was performed. The validation employed the same 4 × 2 MIMO cooperative DF framework considered throughout this work, with QPSK modulation, imperfect CSI ($\sigma_{e}^{2}=0.1$), a rate-1/2 convolutional code (constraint length 7, generator polynomials [171 133]), and hard-decision Viterbi decoding with traceback length 35.

The coded harmful-region metric is defined as

(55) $$\Delta {\mathrm{BER}}_{\mathrm{coded}}={\mathrm{BER}}_{\mathrm{coop},\mathrm{coded}}-{\mathrm{BER}}_{\mathrm{direct},\mathrm{coded}}.$$

**Figure 12:**
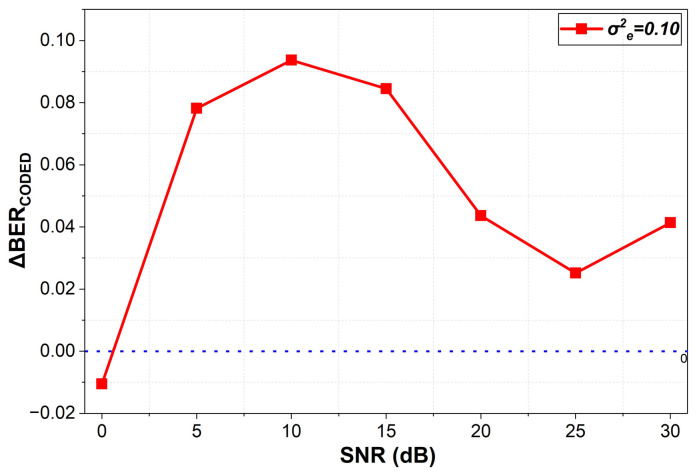
Coded harmful cooperation region for $\sigma_{e}^{2}=0.1$, expressed through $\Delta BER_{\mathrm{coded}}=BER_{\mathrm{coop},\mathrm{coded}}-BER_{\mathrm{direct},\mathrm{coded}}$. Positive values indicate harmful cooperation, whereas negative values indicate beneficial cooperation. The horizontal dashed line indicates the reference boundary at $\Delta BER_{\mathrm{coded}}=0$.

Figure 12 shows that the harmful cooperation phenomenon remains observable even when channel coding is employed. Specifically, $\Delta {\mathrm{BER}}_{\mathrm{coded}}$ remains positive throughout most of the investigated SNR range (approximately 5–30 dB), with values ranging from approximately 0.026 to 0.094. This observation confirms that the harmful cooperation region is not merely an artifact of uncoded BER evaluation but persists under coded transmission as well. The result further supports the interpretation that the phenomenon originates from the underlying impairment mechanisms identified in this work, namely CSI mismatch and relay error propagation.

## 4. Reliability-Aware Relay Activation

### 4.1. Reliability-Aware Relay Activation Under Harmful Cooperation Conditions

The analysis presented in Section 3 demonstrated that cooperative Decode-and-Forward (DF) transmission does not necessarily guarantee performance improvement under imperfect CSI conditions. In particular, under imperfect CSI, the combined effects of residual CSI-induced interference and relay decoding errors may progressively reduce the achievable cooperation gain and eventually lead to the harmful cooperation region, where cooperative transmission underperforms direct MIMO communication.

This degradation is primarily associated with relay decoding reliability. When the relay operates under unfavorable channel conditions, decoding errors become increasingly likely and may subsequently propagate to the destination through the relay transmission path. Since the destination has no explicit knowledge of relay reliability, erroneously detected relay symbols may be combined together with the desired signal, introducing additional structured interference and increasing the overall BER. Similar relay error propagation effects have also been reported in cooperative systems operating under practical impairments [17].

The impact of this behavior is clearly illustrated in Figure 10, where, for severe CSI uncertainty ($\sigma_{e}^{2}=0.1$), cooperative transmission consistently underperforms direct MIMO communication throughout most of the medium-to-high SNR regime. In this region, residual CSI-induced interference together with relay decoding errors progressively dominate system performance, substantially reducing the benefits normally expected from cooperative diversity.

These observations reveal a fundamental limitation of conventional always-on cooperative transmission: the inability to suppress unreliable relay participation under poor channel conditions. Motivated by this limitation, a reliability-aware relay activation mechanism is proposed in order to enable controlled relay participation according to the instantaneous channel conditions.

The proposed approach is based on the observation that relay decoding reliability strongly depends on the effective quality of the source–relay channel. Consequently, suppressing relay transmissions under unfavorable channel conditions can significantly reduce error propagation and improve the overall robustness of cooperative transmission.

To quantify relay reliability, a channel-quality metric based on the normalized Frobenius norm of the effective estimated source–relay channel is employed:

(56) $$\gamma_{SR}={∥{\hat{H}}_{SR}F_{S}∥}_{F}^{2}$$

where ${\hat{H}}_{SR}$ denotes the estimated source–relay channel matrix and $F_{S}$ is the source precoding matrix.

The Frobenius norm is defined as

(57) $${∥A∥}_{F}^{2}=\mathrm{Tr}\left(A^{H}A\right)$$

The proposed relay activation mechanism is directly motivated by the harmful cooperation phenomenon identified in Section 3. Relay participation is therefore controlled according to source–relay link reliability in order to suppress operating conditions where cooperation may become detrimental due to the combined effects of CSI uncertainty and relay error propagation.

Accordingly, $\gamma_{SR}$ represents the normalized energy of the effective source–relay channel and serves as a practical indicator of relay decoding reliability.

Channel-quality-based metrics are widely adopted in relay selection due to their low complexity and practical feasibility [17]. Although more sophisticated reliability metrics could also be considered, the normalized Frobenius norm is selected here because of its simplicity, scalability across antenna configurations, and reliance solely on locally available CSI.

The relay activation rule is defined as

(58) $$\mathrm{Relay}\;\mathrm{active}=\left\{\begin{array}{cc} 1,\; & \gamma_{SR}>\gamma_{\mathrm{th}}\\ 0,\; & \gamma_{SR}\leq \gamma_{\mathrm{th}} \end{array}\right.$$

where $\gamma_{\mathrm{th}}$ denotes the relay activation threshold.

In practice, the proposed reliability metric is computed from the estimated source–relay channel already available at the relay through the standard channel estimation process used for signal detection. Specifically, the relay forms the estimated effective source–relay channel and computes the corresponding channel-quality metric used for relay activation. Consequently, the proposed mechanism does not require additional knowledge of relay BER, outage probability, or channel statistics. Furthermore, the activation decision is based on the estimated effective source–relay channel and therefore inherently reflects the channel estimation uncertainty considered in the imperfect-CSI model.

Although Proposition 1 establishes the existence of SINR saturation under imperfect CSI conditions, it does not directly provide a closed-form relationship between the CSI error variance and the optimal relay activation threshold. Additional threshold-sweep experiments, in which the relay activation threshold was optimized with respect to BER for different CSI error variances and operating SNR values, further indicate that the preferred threshold depends jointly on CSI uncertainty and operating SNR, suggesting that the threshold-selection problem cannot be described solely as a function of CSI error variance. Consequently, the threshold is treated here as a design parameter and evaluated through simulation-based threshold sweeps.

Consequently, the threshold is treated here as a design parameter and selected through simulation-based threshold sweeps. Smaller threshold values increase relay participation and cooperative diversity, whereas larger threshold values improve transmission reliability by suppressing unreliable relay forwarding.

From a reliability perspective, the proposed activation mechanism suppresses relay participation when the effective source–relay channel quality becomes insufficient to guarantee reliable decoding. Consequently, the activation threshold acts as a reliability filter that reduces the probability of relay error propagation under imperfect CSI conditions.

For relatively small threshold values, the relay remains active for most channel realizations, closely approximating conventional always-on cooperative transmission. In this regime, the system benefits from increased cooperative diversity; however, unreliable relay decoding becomes more likely under imperfect CSI conditions, thereby increasing the probability of relay error propagation.

Conversely, as the activation threshold increases, relay participation becomes progressively more selective. This reduces the likelihood of unreliable relay forwarding and suppresses CSI-induced error propagation. Nevertheless, excessively large threshold values may deactivate the relay for a significant fraction of channel realizations, effectively reducing the achievable cooperation gain and causing the system behavior to gradually approach direct MIMO transmission.

**Figure 13:**
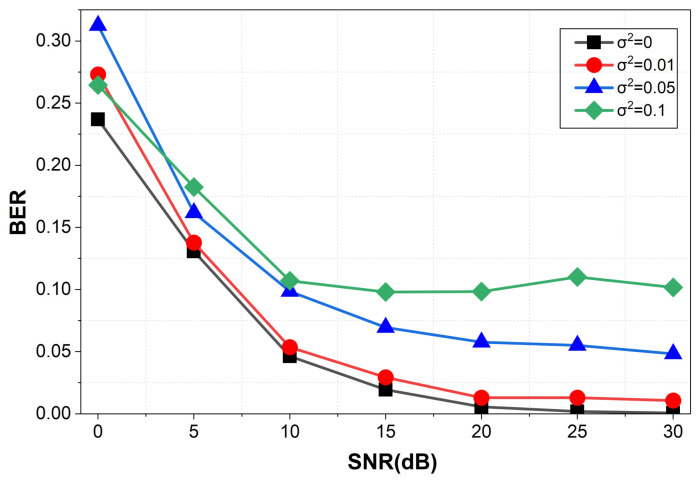
BER performance of reliability-aware cooperative DF ($\gamma_{\mathrm{th}}=1$) for different $\sigma_{e}^{2}$.

As an initial operating point, a threshold value of $\gamma_{\mathrm{th}}=1$ is considered in order to illustrate the basic behavior of reliability-aware relay activation under imperfect CSI conditions. This relatively low threshold allows frequent relay participation while still suppressing severely unreliable channel realizations. The impact of the proposed mechanism is illustrated in Figure 13, where BER reduction together with partial mitigation of the high-SNR error floor can be observed, particularly under moderate and severe CSI uncertainty conditions.

Overall, the proposed mechanism acts as a low-complexity reliability filter that adaptively suppresses unreliable relay participation under poor channel conditions. From a practical standpoint, the approach is particularly attractive because it requires only local CSI, introduces no additional signaling overhead, and maintains very low implementation complexity.

At the same time, the results indicate that the effectiveness of reliability-aware cooperation strongly depends on the selection of the activation threshold. This observation motivates the threshold optimization analysis presented in the following subsection.

### 4.2. Threshold Selection and Optimization

The previous results demonstrated that the effectiveness of the proposed reliability-aware relay activation mechanism strongly depends on the selection of the activation threshold. Since the threshold directly controls relay participation, it also determines the balance between cooperative diversity gain and relay-induced error propagation.

For low threshold values, the relay remains active for almost all channel realizations, resulting in activation rates close to unity. Under these conditions, the system behavior closely resembles conventional always-on cooperative transmission. Although cooperation remains continuously available, unreliable relay transmissions are also frequently forwarded, leading to persistent error propagation and noticeable BER degradation.

As the activation threshold increases, relay participation becomes progressively more selective. This reduces the number of unreliable relay transmissions and consequently improves BER performance. At the same time, however, excessive suppression of relay activity reduces the diversity benefits provided by cooperative transmission. The resulting behavior highlights the inherent trade-off between cooperative diversity gain and relay-induced interference suppression [17].

Between these two extremes, an intermediate operating region emerges in which relay participation becomes sufficiently selective to suppress unreliable transmissions while still preserving the advantages of cooperative diversity. Optimal performance is observed in this region for relay activation rates of approximately 40–50%, as summarized in Table 3.

The threshold selection analysis is presented for a moderate CSI error variance ($\sigma_{e}^{2}=0.05$), where both cooperative diversity gain and relay error propagation effects are simultaneously observable. This operating regime provides a clear view of the trade-off between diversity enhancement and relay-induced interference, which becomes less distinguishable under severe CSI uncertainty conditions.

**Figure 14:**
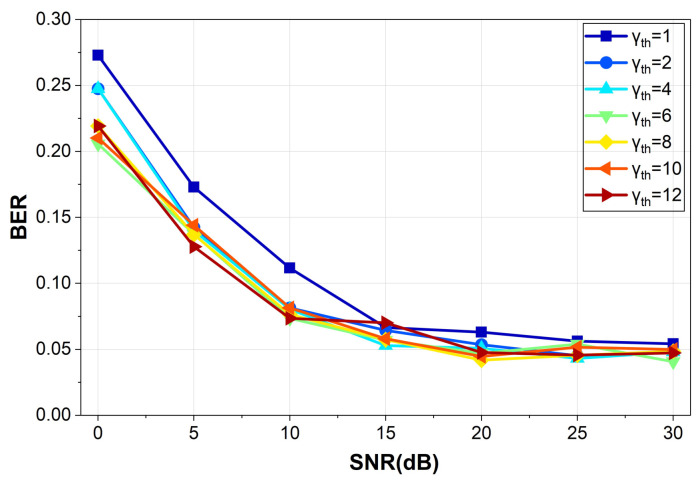
BER vs. SNR for different activation thresholds $\gamma_{\mathrm{th}}$ ($\sigma_{e}^{2}=0.05$).

The balanced activation behavior achieved within the intermediate threshold region is also reflected in Figure 14, where the minimum BER performance is observed. In contrast, for very high threshold values, the relay is only rarely activated, with activation rates falling below 20%. Under these conditions, the system behavior gradually approaches direct MIMO transmission, resulting in reduced cooperative gain and slightly increased BER relative to the optimal operating region.

The previous observations further indicate that no single threshold value can provide optimal performance across all SNR and CSI regimes. Consequently, the activation threshold must adapt to the operating conditions of the system.

In this work, the threshold is determined empirically through exhaustive evaluation over a range of candidate values, selecting the threshold that minimizes BER for each operating condition. As illustrated in Figure 14, the optimal threshold varies significantly depending on both the SNR level and the degree of CSI uncertainty.

For severe CSI uncertainty, the optimal thresholds are generally found within the intermediate operating region, where the balance between cooperative diversity gain and relay-induced error propagation is best maintained. In contrast, for lower CSI uncertainty levels, more frequent relay activation becomes beneficial since the probability of relay decoding errors is substantially reduced.

These observations confirm that threshold selection constitutes a non-monotonic optimization problem. More importantly, the optimized relay activation strategy substantially mitigates the harmful cooperation region identified in Section 3, confirming that selective relay participation can effectively recover the advantages of cooperative transmission under imperfect CSI conditions.

Overall, the results demonstrate that the best system performance is achieved not through continuous cooperation, but through adaptive relay participation based on transmission reliability and channel conditions.

### 4.3. Performance Recovery Through Reliability-Aware Cooperation

To further evaluate the effectiveness of the proposed approach, a final comparison is performed between three transmission strategies: direct MIMO transmission, cooperative DF with always-on relay operation, and reliability-aware cooperative transmission with optimized threshold selection.

The results demonstrate that conventional always-on cooperative transmission becomes highly vulnerable under imperfect CSI conditions due to persistent relay error propagation and residual CSI-induced interference. In particular, as the SNR increases, relay decoding errors continue to propagate through the cooperative transmission path, progressively reducing the achievable cooperation gain and leading to substantial BER degradation in the medium-to-high SNR regime.

In contrast, the proposed reliability-aware strategy significantly improves transmission robustness by selectively suppressing unreliable relay participation. By activating the relay only under sufficiently reliable source–relay channel conditions, the proposed mechanism effectively reduces relay-induced interference and limits the propagation of erroneously detected relay symbols.

**Figure 15:**
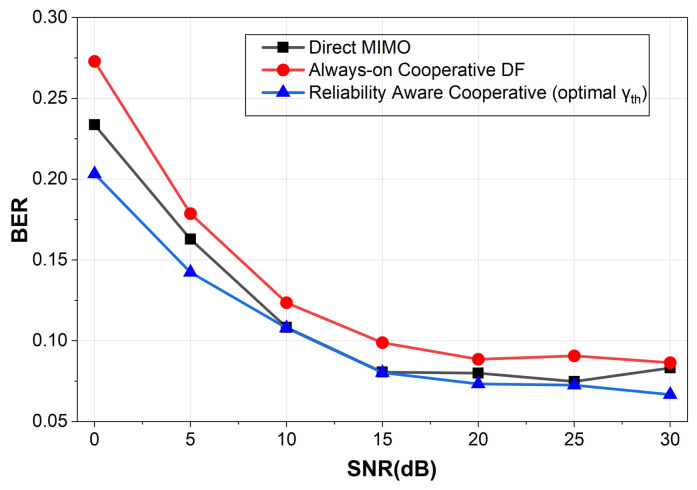
BER comparison of direct MIMO, always-on cooperative DF and reliability-aware cooperative transmission for $\sigma_{e}^{2}=0.1$.

As illustrated in Figure 15, the proposed reliability-aware scheme substantially outperforms conventional always-on cooperative transmission and consistently achieves lower BER than direct MIMO transmission in the medium-to-high SNR regime. These results confirm that selective relay participation can effectively restore the advantages of cooperative diversity while simultaneously mitigating the detrimental effects introduced by imperfect CSI and relay decoding errors.

Furthermore, the proposed approach exhibits improved stability in the high-SNR regime, avoiding the severe BER degradation observed under conventional always-on cooperative transmission. Most importantly, the results indicate that selective relay participation can transform cooperative transmission from a detrimental operating mode into a beneficial one under severe CSI uncertainty conditions.

While always-on cooperation amplifies relay-induced interference and error propagation, the proposed reliability-aware mechanism preserves the advantages of cooperative diversity by suppressing unreliable relay forwarding. Consequently, the harmful cooperation region identified in the previous analysis is substantially reduced through adaptive relay participation.

Overall, the results confirm that reliability-aware relay activation constitutes an effective low-complexity strategy for mitigating harmful cooperation behavior under imperfect CSI conditions while preserving the achievable gains of cooperative transmission. From a practical perspective, the proposed approach is particularly attractive because it requires only local CSI information, introduces minimal implementation complexity, and can be readily integrated into practical cooperative MIMO systems operating under realistic channel uncertainty conditions.

To further support the low-complexity claim of the proposed reliability-aware relay activation mechanism, a computational complexity assessment was performed. Specifically, relay activation requires the evaluation of the reliability metric

(59) $$\gamma_{SR}={∥{\hat{H}}_{SR}F_{S}∥}_{F}^{2}$$

followed by a simple threshold comparison.

In contrast, the dominant computational burden of both conventional and reliability-aware cooperative DF schemes arises from the MIMO detection stage, which requires pseudo-inverse operations. Therefore, the proposed activation mechanism introduces only a minor additional processing cost relative to the signal detection operations already required by the receiver.

To quantify this overhead, a runtime comparison was performed in MATLAB R2018b using 10^6^ evaluations. The relay activation metric required 0.279 s, whereas a pseudo-inverse operation required 14.296 s. Thus, the runtime associated with relay activation corresponded to only 1.95% of that required for the pseudo-inverse computation. Equivalently, the pseudo-inverse operation was approximately 51 times more computationally demanding than the relay activation metric.

These results confirm that the proposed reliability-aware relay activation mechanism can be implemented with negligible computational overhead while providing improved robustness under imperfect CSI conditions.

## 5. Antenna Scaling and System-Level Robustness

The previous sections demonstrated that cooperative transmission under imperfect CSI is strongly affected by relay error propagation and residual interference. In addition to relay activation strategies, system dimension also plays an important role in determining the overall robustness of cooperative MIMO transmission. To investigate this effect, the impact of antenna scaling on system robustness is analyzed in this section.

To quantitatively evaluate the sensitivity of the system to channel estimation errors, the CSI sensitivity metric is defined as

(60) $$S_{\mathrm{CSI}}=\frac{{\mathrm{BER}}_{\sigma_{e}^{2}}-{\mathrm{BER}}_{0}}{{\mathrm{BER}}_{0}}$$

where ${\mathrm{BER}}_{0}$ denotes the BER obtained under the baseline case $\sigma_{e}^{2}=0$, while ${\mathrm{BER}}_{\sigma_{e}^{2}}$ corresponds to the BER under imperfect CSI conditions.

The relative formulation is selected in order to normalize the impact of CSI uncertainty with respect to the baseline system performance, thereby enabling fair robustness comparison across different antenna configurations and transmission schemes. Since extremely small BER values may lead to numerical instability in relative sensitivity evaluation, a stabilized version of the metric is additionally considered:

(61) $$S_{\mathrm{CSI}}^{\mathrm{safe}}=\frac{{\mathrm{BER}}_{\sigma_{e}^{2}}-{\mathrm{BER}}_{0}}{\max({\mathrm{BER}}_{0},\epsilon )}$$

where ϵ denotes a small BER floor introduced to avoid instability when ${\mathrm{BER}}_{0}\to 0$.

Based on this metric, the robustness gain is further defined as

(62) $$G_{R}=S_{\mathrm{CSI}}^{\mathrm{direct}}-S_{\mathrm{CSI}}^{\mathrm{coop}}$$

providing a direct comparison between the robustness of direct and cooperative transmission schemes. Positive values of $G_{R}$ indicate that cooperative transmission is more resilient to CSI uncertainty, whereas negative values imply higher sensitivity relative to direct MIMO communication.

**Figure 16:**
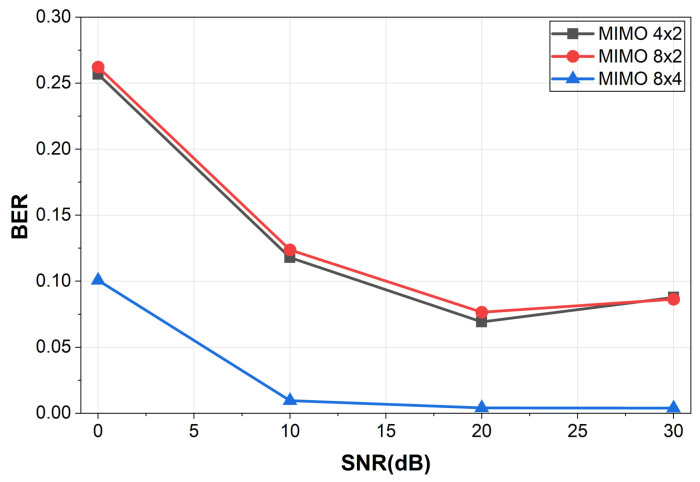
BER comparison across antenna configurations under imperfect CSI ($\sigma_{e}^{2}=0.1$).

To examine the effect of system dimension, simulations are performed for multiple MIMO configurations (4×2, 8×2, and 8×4), while maintaining a fixed number of transmitted streams and identical system parameters. The corresponding BER versus SNR performance is illustrated in Figure 16, where each curve corresponds to a different antenna configuration.

The results clearly show that increasing the system dimension leads to substantial BER improvement, particularly in the moderate-to-high SNR regime and under severe CSI uncertainty conditions. This improvement can largely be attributed to the increased spatial diversity and improved conditioning of the effective channel matrix, both of which reduce the impact of noise amplification and channel estimation errors [6,7].

The pronounced BER improvement observed for the 8 × 4 configuration can be further explained through the conditioning of the effective estimated channel matrix

(63) $$\hat{G}=\hat{H}F,$$

where $\hat{H}$ denotes the estimated channel matrix and F is the precoding matrix. The channel conditioning was quantified through the condition number

(64) $$\kappa \left(\hat{G}\right)=\frac{\sigma_{\max}\left(\hat{G}\right)}{\sigma_{\min}\left(\hat{G}\right)},$$

where smaller values indicate a better-conditioned detection problem. Under severe CSI uncertainty ($\sigma_{e}^{2}=0.1$), the median condition number was approximately 2.82–2.96 for the 4 × 2 configuration and 2.83–3.02 for the 8 × 2 configuration, whereas it decreased to approximately 1.86–1.87 for the 8 × 4 configuration. This improvement originates from the overdetermined effective channel structure of the 8 × 4 case ($N_{r}=4>N_{s}=2$), which provides additional receive-side spatial redundancy. The observed behavior is therefore primarily associated with the receive-side dimensionality increase ($N_{r}=4$) rather than with the increase in the number of transmit antennas alone. Consequently, pseudo-inverse noise enhancement is reduced and the impact of CSI perturbations becomes less pronounced, leading to significantly improved robustness against channel estimation errors.

**Figure 17:**
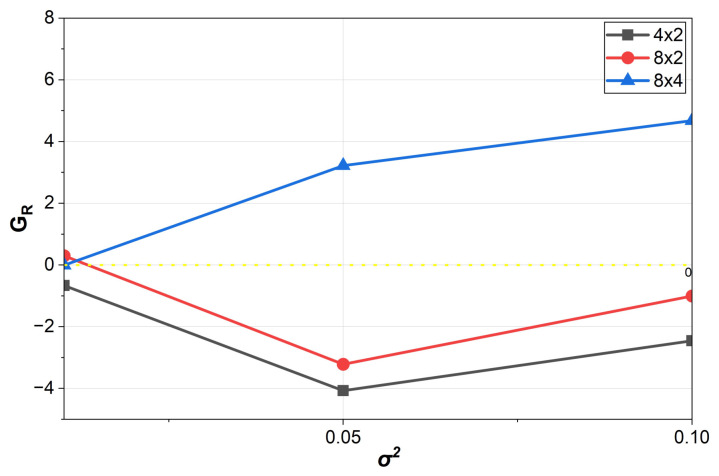
Robustness gain versus CSI error variance for different antenna configurations at 20 dB. The metric is defined as $G_{R}^{\mathrm{safe}}=S_{\mathrm{CSI},\mathrm{direct}}^{\mathrm{safe}}-S_{\mathrm{CSI},\mathrm{cooperative}}^{\mathrm{safe}}$. Positive values indicate robustness improvement, whereas negative values indicate robustness degradation. The horizontal dashed line indicates the reference boundary at $G_{R}=0$.

The effect of antenna scaling is further reflected in the robustness gain metric shown in Figure 17, where a clear system-dependent trend can be observed. For smaller configurations, such as 4×2 and 8×2, the robustness gain remains predominantly negative, indicating that cooperative transmission is more sensitive to CSI imperfections due to relay-induced error propagation. In contrast, for the larger 8×4 configuration, the robustness gain becomes positive over a wide range of CSI error values, demonstrating that cooperative transmission can become more robust than direct MIMO under sufficiently large system dimensions.

This robustness transition highlights the strong dependence of cooperative transmission performance on system dimension, a behavior that has also been observed in large-scale MIMO systems [6,7]. The underlying reason lies in the spatial averaging effect introduced by higher-dimensional antenna configurations. As the number of antennas increases, the effective channel matrix becomes better conditioned, while the impact of individual channel estimation errors and relay decoding errors is distributed across multiple spatial dimensions. Consequently, the variance of the effective interference is reduced, leading to improved detection reliability.

In this regime, the diversity gain provided by larger antenna configurations can partially compensate for the degradation introduced by relay error propagation, allowing cooperative transmission to outperform direct MIMO in terms of robustness to CSI uncertainty [6,7].

Nevertheless, antenna scaling alone is insufficient to completely eliminate performance degradation under imperfect CSI. Even for larger configurations, residual sensitivity persists, particularly in the high-SNR regime where the system progressively becomes interference-limited and BER saturation appears due to residual CSI-induced interference [3,18]. In addition, the robustness gain is not uniform across all CSI error levels, indicating that the benefits of antenna scaling remain strongly dependent on the operating conditions.

Overall, these findings demonstrate that increasing the system dimension improves robustness but does not fundamentally resolve the limitations of cooperative transmission under realistic conditions. Consequently, achieving robust performance requires a joint design approach that combines spatial diversity with reliability-aware relay activation, as introduced in Section 4. Such a combined strategy provides both structural robustness through antenna scaling and adaptive robustness through selective relay participation, offering a more effective framework for cooperative MIMO systems operating under imperfect CSI conditions.

## 6. Discussion and Insights

The results presented throughout this work demonstrate that cooperative MIMO transmission is not inherently beneficial under realistic operating conditions. Although Decode-and-Forward relaying can provide substantial diversity gains under ideal assumptions, its effectiveness is significantly reduced in the presence of CSI uncertainty and relay decoding errors. Under such conditions, the expected cooperative gains may be offset by CSI-induced residual interference together with relay error propagation, revealing a fundamental limitation of conventional always-on cooperative transmission [15,17].

A particularly important observation is that the effectiveness of cooperation strongly depends on the operating SNR regime. In the low-SNR region, where thermal noise remains the dominant impairment, cooperative transmission provides the expected diversity benefits and improves BER performance. As the SNR increases, however, the system progressively transitions toward an interference-limited regime in which residual CSI-induced interference becomes dominant. In this regime, the benefits of cooperation are substantially reduced, leading to the emergence of the harmful cooperation region where cooperative transmission underperforms direct MIMO communication. This behavior is consistent with the performance saturation previously reported for imperfect CSI systems operating at high SNR [3].

The analysis further indicates that the observed degradation is primarily driven by CSI uncertainty and subsequently amplified by relay error propagation. In cooperative systems, relay decoding errors introduce structured interference that cannot be effectively mitigated by increasing the transmit power. As a result, cooperative transmission becomes increasingly sensitive to practical impairments, particularly in small-scale MIMO configurations where the available spatial diversity remains limited.

To address these limitations, a reliability-aware relay activation mechanism was introduced in order to selectively control relay participation according to channel conditions. The proposed approach effectively reduces error propagation by suppressing unreliable relay transmissions, thereby improving BER performance under imperfect CSI conditions. At the same time, the results indicate that reliability-aware activation does not completely eliminate the effects of CSI uncertainty, but instead establishes a controlled trade-off between cooperative gain and transmission robustness.

In addition to relay reliability, the impact of system dimension was also investigated. The results reveal that the robustness of cooperative transmission improves significantly as the antenna configuration increases. While small-scale cooperative systems remain highly sensitive to CSI imperfections, larger antenna configurations benefit from increased spatial diversity and improved channel conditioning, both of which reduce the relative impact of estimation errors and relay error propagation [6]. Nevertheless, antenna scaling alone is insufficient to fully overcome the limitations introduced by imperfect CSI, particularly in the high-SNR regime where interference-limited behavior persists.

Overall, the findings of this work, summarized in Table 4, suggest that effective cooperative MIMO design under realistic conditions requires the joint consideration of CSI accuracy, relay reliability and system dimension. In particular, adaptive relay activation mechanisms are essential for avoiding performance degradation caused by unreliable relay transmissions, while spatial diversity can significantly improve system robustness. Consequently, practical cooperative MIMO systems should be designed through a balanced optimization of cooperation gain, robustness, and implementation complexity.

## 7. Conclusions

This work investigated the performance of cooperative MIMO systems under imperfect CSI and relay decoding conditions, with particular emphasis on the impact of channel estimation errors and relay decoding imperfections. The results demonstrated that, although cooperative transmission can provide substantial performance gains under ideal assumptions, its effectiveness degrades significantly in the presence of imperfect CSI.

More specifically, CSI uncertainty introduces residual interference that leads to performance saturation in the high-SNR regime, while relay decoding errors further amplify this degradation through error propagation. As a consequence, cooperative transmission may underperform direct MIMO communication within specific SNR operating regions, leading to the emergence of the harmful cooperation region. These findings demonstrate that cooperative transmission is not inherently beneficial under practical CSI limitations and therefore requires reliability-aware control mechanisms.

To mitigate these limitations, a reliability-aware relay activation mechanism was proposed, enabling selective relay participation according to channel quality. The obtained results showed that the proposed approach effectively reduces relay error propagation and improves BER performance, particularly in the medium-to-high SNR regime where conventional always-on cooperation becomes ineffective. Nevertheless, residual sensitivity to CSI uncertainty remains present, indicating that adaptive relay activation can alleviate, but not completely eliminate, the effects of imperfect channel knowledge.

In addition, the impact of antenna scaling was investigated, revealing that system dimension plays a critical role in cooperative transmission robustness. Larger antenna configurations benefit from increased spatial diversity and improved channel conditioning, both of which reduce the relative impact of CSI errors and relay-induced interference. However, antenna scaling alone is insufficient to fully overcome the limitations introduced by imperfect CSI and relay decoding errors. The analytical interpretation developed throughout this work further demonstrated that the observed BER saturation and harmful cooperation behavior originate from the interference-limited nature of cooperative transmission under imperfect CSI conditions. Overall, the findings of this work demonstrate that robust cooperative MIMO design under imperfect CSI conditions requires the joint consideration of CSI accuracy, relay reliability, adaptive relay participation, and system dimension.

A limitation of the present study is that relay placement effects were not explicitly considered. All communication links were assumed to experience statistically identical average channel conditions in order to isolate the impact of CSI uncertainty and relay error propagation. The adopted assumptions were intentionally selected to enable a clear characterization of the Harmful Cooperation Region without introducing additional topology-dependent effects. Future work will therefore investigate relay-location-dependent behavior, distance-dependent propagation effects and topology-aware cooperative strategies in order to further evaluate and extend the applicability of the proposed reliability-aware framework.

## Figures and Tables

**Table 1:** Structured Comparison of Related Work and Positioning of This Study.

Category	Representative Works	Key Focus	Limitation
MIMO Detection under Imperfect CSI	[10,11,12]	Robust detection	No cooperative analysis
Cooperative MIMO Systems	[13,15,16,17,18,33]	Relay-assisted transmission	No joint CSI–relay effects
Virtual/Collaborative MIMO	[21,22,23,24,25,26,27]	Terminal cooperation	Cooperation assumed beneficial
Relay Selection & DF Relaying	[4,28,29,30]	Relay selection	Cooperation assumed beneficial
Practical Impairments & Modern Systems	[5,34,35]	Hardware impairments	No degradation-regime analysis
Theoretical Foundations	[2,3,6,32]	Performance limits	Not extended to cooperative systems
This Work	Not applicable	Harmful cooperation region and reliability-aware relay activation	Joint CSI uncertainty and relay decoding error propagation

**Table 2:** Simulation Parameters.

Parameter	Value	Description
Simulator	Vienna SLS	System-level framework
Transmission Scheme	Spatial Multiplexing ($N_{s}=2$)	Two spatial streams
Transmit/Receive Antennas	$\left(N_{t},N_{r}\right)=\left(4,2\right),\left(8,2\right),\left(8,4\right)$	Evaluated configurations
Relay Protocol	Decode-and-Forward (DF)	Half-duplex relaying
Channel Model	Rayleigh fading	i.i.d. fading
Precoding	Semi-unitary DFT precoding	Precoding matrix F
CSI Model	$\hat{H}=H+E$	Estimation error model
CSI Error Variance	$\sigma_{e}^{2}\in \left\{0,0.01,0.05,0.1\right\}$	CSI uncertainty
Detection Method	ZF/MMSE	Linear detection
Noise Model	AWGN: $\mathrm{CN}(0,\sigma_{n}^{2}I)$	Gaussian noise
Combining Scheme	Selection Combining	Gain-based selection
SNR Range	0–30 dB	Operating range
Modulation	QPSK	Gray mapping
Threshold ($\gamma_{\mathrm{th}}$)	Swept	Relay activation criterion

**Table 3:** Impact of Threshold Selection on Relay Activation and BER Performance.

Threshold ($\gamma_{\mathrm{th}}$)	Activation Rate	BER (Mid–High SNR)	Interpretation
1	∼0.98–0.99	∼0.06–0.08	Always-on cooperation
2–4	∼0.80–0.90	∼0.05–0.07	High relay activity
6–8	∼0.40–0.50	∼0.04–0.05	Optimal regime
10–12	∼0.10–0.20	∼0.05–0.07	Near-direct operation

**Table 4:** Analytical Interpretation of the Main Simulation Observations.

Simulation Observation	Analytical Interpretation
BER saturation under imperfect CSI	Residual CSI-induced interference scales with signal power, causing SINR saturation at high SNR.
Cooperative DF underperforms direct MIMO in the harmful region	Relay error propagation may outweigh cooperative diversity gains.
Reliability-aware relay activation improves BER	Selective relay activation suppresses relay-induced interference.
Larger antenna configurations improve robustness	Larger MIMO dimensions improve channel conditioning and reduce CSI sensitivity.

## Data Availability

The simulation data supporting the findings of this study are available from the corresponding author upon reasonable request.
